# Phenotyping Post-COVID Pain as a Nociceptive, Neuropathic, or Nociplastic Pain Condition

**DOI:** 10.3390/biomedicines10102562

**Published:** 2022-10-13

**Authors:** César Fernández-de-las-Peñas, Jo Nijs, Randy Neblett, Andrea Polli, Maarten Moens, Lisa Goudman, Madhura Shekhar Patil, Roger D. Knaggs, Gisele Pickering, Lars Arendt-Nielsen

**Affiliations:** 1 Department of Physical Therapy, Occupational Therapy, Rehabilitation and Physical Medicine, Universidad Rey Juan Carlos, 28922 Alcorcón, Spain; 2 Center for Neuroplasticity and Pain (CNAP), SMI, Department of Health Science and Technology, Faculty of Medicine, Aalborg University, 9220 Aalborg, Denmark; 3 Pain in Motion Research Group (PAIN), Department of Physiotherapy, Human Physiology and Anatomy, Faculty of Physical Education & Physiotherapy, Vrije Universiteit Brussel, 1050 Brussels, Belgium; 4 Chronic Pain Rehabilitation, Department of Physical Medicine and Physiotherapy, University Hospital Brussels, 1050 Brussels, Belgium; 5 Unit of Physiotherapy, Department of Health and Rehabilitation, Institute of Neuroscience and Physiology, Sahlgrenska Academy, University of Gothenburg, 41390 Gotëborg, Sweden; 6 PRIDE Research Foundation, Dallas, TX 75235, USA; 7 Research Foundation–Flanders (FWO), 1000 Brussels, Belgium; 8 Laboratory of Clinical Epigenetics, Department of Public Health and Primary Care, Centre for Environment & Health, KU Leuven, 3000 Leuven, Belgium; 9 Department of Neurosurgery, Universitair Ziekenhuis Brussel, 1090 Brussels, Belgium; 10 STIMULUS Research Group (reSearch and TeachIng neuroModULation Uz bruSsel), Vrije Universiteit Brussel, Laarbeeklaan 103, 1090 Brussels, Belgium; 11 Center for Neurosciences (C4N), Vrije Universiteit Brussel, 1050 Brussels, Belgium; 12 Department of Radiology, Universitair Ziekenhuis Brussel, Laarbeeklaan 101, 1090 Brussels, Belgium; 13 Clinical Pharmacy Practice, School of Pharmacy, University of Nottingham, Nottingham NG7 2RD, UK; 14 Plateforme d’Investigation Clinique, University Hospital Clermont-Ferrand, Inserm CIC 1405, F-63000 Clermont-Ferrand, France; 15 Department of Medicine and Clinical Pharmacology, University Clermont Auvergne, Inserm 1107, F-63000 Clermont-Ferrand, France; 16 Department of Medical Gastroenterology, Mech-Sense, Aalborg University Hospital, 9000 Aalborg, Denmark

**Keywords:** COVID-19, post-COVID, nociplastic pain, neuropathic pain, musculoskeletal pain, precision medicine, peripheral sensitization, central sensitization, nociceptive

## Abstract

Pain after an acute Severe Acute Respiratory Syndrome Coronavirus 2 (SARS-CoV-2) infection and coronavirus disease 2019 (COVID-19) condition (post-COVID pain) is becoming a new healthcare emergency. Precision medicine refers to an evidence-based method of grouping patients based on their diagnostic/symptom presentation and then tailoring specific treatments accordingly. Evidence suggests that post-COVID pain can be categorized as nociceptive (i.e., pain attributable to the activation of the peripheral receptive terminals of primary afferent neurons in response to noxious chemical, mechanical, or thermal stimuli), neuropathic (i.e., pain associated with a lesion or disease of the somatosensory nervous system and limited to a “neuroanatomically plausible” distribution of the system), nociplastic (i.e., pain arising from altered nociception despite no clear evidence of actual or threatened tissue damage causing the activation of peripheral nociceptors or evidence for disease or lesion of the somatosensory system causing the pain), or mixed type (when two pain phenotypes co-exist). Each of these pain phenotypes may require a different treatment approach to maximize treatment effectiveness. Accordingly, the ability to classify post-COVID pain patients into one of these phenotypes would likely be critical for producing successful treatment outcomes. The 2021 International Association for the Study of Pain (IASP) clinical criteria and grading system provide a framework for classifying pain within a precision pain medicine approach. Here we present data supporting the possibility of grouping patients with post-COVID pain into pain phenotypes, using the 2021 IASP classification criteria, with a specific focus on nociplastic pain, which is probably the primary mechanism involved in post-COVID pain. Nociplastic pain, which is usually associated with comorbid symptomology (e.g., poor sleep quality, fatigue, cognitive–emotional disturbances, etc.) and is considered to be more difficult to treat than other pain types, may require a more nuanced multimodal treatment approach to achieve better treatment outcomes.

## 1. Introduction

The worldwide outbreak induced by the Severe Acute Respiratory Syndrome Coronavirus 2 (SARS-CoV-2), the virus responsible for the coronavirus disease 2019 (COVID-19), has dramatically changed healthcare systems over the last few years. After millions of infections, healthcare professionals are now confronted with another associated crisis—the development or persistence of symptoms after the acute phase of SARS-CoV-2 infection, a condition called long COVID [1] or post-COVID-19 [2]. More than 100 symptoms have been described, affecting multiple systems, e.g., cardiovascular, neurological, respiratory, and musculoskeletal [3]. In fact, several meta-analyses have observed that almost 50% of COVID-19 survivors exhibit a plethora of lingering symptoms lasting for weeks or months [4,5,6] and up to one year after infection [7,8,9]. A recent systematic review investigating multiple post-COVID symptoms identified that 20% of COVID-19 survivors reported post-COVID pain at different follow-up periods during the first year after infection [10]. Other studies, which have specifically investigated post-COVID pain symptoms, found prevalence rates of up to 60% of COVID-19 survivors [11,12,13,14]. Accordingly, post-COVID pain could be underestimated and undertreated if not properly identified and classified.

Precision medicine refers to an evidence-based method of subgrouping patients, based on diagnostic and symptom presentation, and then tailoring specific treatments to individual patient phenotypes based on the prognosis for positive treatment outcomes and susceptibility to negative outcomes [15]. Three major pain phenotypes have been identified (i.e., nociceptive, neuropathic, and nociplastic pain). Accordingly, subgrouping patients with post-COVID pain could result in the most successful treatment outcomes. However, discrimination between these phenotypes can be challenging, since patients can fit into more than one phenotype (e.g., mixed-type); since identifying one type (i.e., neuropathic) does not exclude another (i.e., nociplastic) [16].

Nociplastic pain has been defined by the International Association for the Study of Pain (IASP) as “pain that arises from altered nociception despite no clear evidence of actual or threatened tissue damage causing the activation of peripheral nociceptors or evidence for disease or lesion of the somatosensory system causing the pain”; it was introduced as a third mechanistic pain descriptor in addition to nociceptive and neuropathic pain [17]. Though it has become well-established in recently published literature, this definition of nociplastic pain has also raised questions [18]. One challenge with this definition is that it relies on a determination of altered pain processing (e.g., pain hypersensitivity). Currently, no gold standard exists for determining whether an individual patient is experiencing a normal or heightened pain response. In 2021, the IASP released the first set of clinical criteria and a grading system for identifying nociplastic pain [19]. These criteria are comprehensive, robust, properly developed, and have a high potential to be useful for clinicians [20]. Although primarily developed for patients with chronic pain of musculoskeletal origin, the IASP nociplastic criteria can be also applied to individuals with post-COVID pain.

It should be noted that some individuals who were infected with COVID-19 had a previous history of chronic pain. It stands to reason that they might respond to the virus differently and may be more susceptible to long COVID pain than individuals without previous chronic pain conditions. In such a scenario, premorbid pain could lead to a worse prognosis of post-COVID pain and represents a risk factor for future development of the nociplastic pain phenotype. The millions of individuals infected by SARS-COV-2 may provide a unique opportunity to investigate pain after a viral infection, as the pain features may be similar to other previous viral infections (not yet investigated as COVID-19) and, therefore, knowledge can be transferred between conditions.

Being able to identify individuals with a nociplastic pain phenotype has the potential to improve precision pain medicine practices in musculoskeletal pain conditions [21]. In the current narrative review, an international group of experts in chronic pain propose a clinical rationale for the application of the 2021 IASP clinical criteria to identify nociplastic pain in the growing population of COVID-19 survivors with post-COVID pain so that the most effective treatment approaches can be provided. Proper distinction among pain phenotypes is important because neuropathic and nociplastic pain are considered to be more difficult to treat than pure nociceptive (e.g., musculoskeletal) pain. In addition, some treatment approaches for nociceptive pain disorders, which have a high probability for success with this phenotype, could be ineffective or even exacerbate symptoms in patients with the other phenotypes, especially those with nociplastic pain. This paper will help clinicians to potentially classify individuals with post-COVID pain symptoms into one pain phenotype and will also propose the clinical reasoning for the treatment of post-COVID pain patients according to the identified pain phenotype.

## 2. Phenotyping Post-COVID Pain

### 2.1. Nociceptive Pain

Nociceptive pain is defined as pain attributable to the activation of the peripheral receptive terminals of primary afferent neurons in response to noxious chemical, mechanical, or thermal stimuli [22]. Clinically, the term nociceptive pain can be used when a pain response is proportional to the nociceptive input [23]. Current theories propose that SARS-CoV-2 cytokine- and interleukin-associated storms may lead to the sensitization of pain pathways [24,25]. Accordingly, it is possible that patients with post-COVID pain can exhibit nociceptive pain features.

D’Souza et al. observed that the most common type of post-COVID pain described by patients themselves on social media resembles a musculoskeletal/nociceptive pain phenotype [26]. In fact, a large cohort study reported that post-COVID pain in previously hospitalized COVID-19 survivors was of musculoskeletal origin in 45% of subjects at eight [27] and twelve [28] months after hospitalization. These authors stated that 30% of COVID-19 survivors with post-COVID pain reported the presence of symptoms solely in localized body areas (e.g., neck, shoulder, elbow, knee, or hip), another 30% exhibited pain in the extremities, and 20% in the spine, whereas the remaining 20% had widespread symptoms [27,28]; however, these authors did not differentiate between pain from muscular or articular origin.

The presence of post-COVID joint pain has reportedly ranged from 8% to 55% [29]. The most frequently involved joints are the knee (38%), followed by hand (25%) and shoulder (19%) [30]. Post-COVID articular pain can be treated with non-steroidal anti-inflammatory drugs (NSAIDs) and local steroids with good results [31], suggesting that these localized, post-COVID arthritic pain symptoms exhibit nociceptive features. Nevertheless, it is important to consider that a large proportion of subjects with post-COVID joint or muscle pain exhibit generalized/widespread symptoms [32]. This generalized pain pattern may be related to the hypothesis that local connective tissue damage caused by SARS-CoV-2 in patients with joint hypermobility can lead to widespread symptomatology [33]. In fact, a widespread pain pattern is indicative of nociplastic pain, which could be present in a subgroup of joint and musculoskeletal pain patients.

### 2.2. Neuropathic Pain

The IASP has proposed the following definition of neuropathic pain: 1. a lesion or disease of the somatosensory nervous system (i.e., central or peripheral nervous system) is identifiable; 2. pain is limited to a ”neuroanatomically plausible” distribution of the system; and 3. supported by clinical examination findings as well as imaging and/or laboratory findings [34]. The neuro-invasive potential associated with SARS-CoV-2 infection, related to the high expression of angiotensin-converting enzyme 2 (ACE2) receptors detected in nervous system cells, including neurons and microglia within the spinal dorsal horn [35], could explain the development of neuropathic pain in COVID-19 survivors. However, the exact role of ACE2 receptors in peripheral small-fiber sensory neurons is still unknown [36].

The presence of neuropathic pain has been well-documented in some individuals with long COVID, e.g., by developing post-herpetic neuralgia, trigeminal neuralgia, or brachial plexopathy [37]. These types of neuropathic pain sequelae have also been seen in other viruses such as Epstein–Barr virus, cytomegalovirus, influenza A virus, or Zika [38]. A cohort study found that 25% of patients with post-COVID pain self-reported neuropathic pain symptoms [39]. Using the Self-Report Leeds Assessment of Neuropathic Symptoms (S-LANSS) [40], Herrero-Montes et al. found that 20% of patients with post-COVID pain fulfilled the criteria (S-LANSS ≥ 12 points) for susceptible neuropathic features [41]. The use of the S-LANSS and PainDETECT to determine the prevalence of neuropathic features has produced slightly different results—using the S-LANSS cutoff score of ≥12 points, 26% of COVID-19 survivors with post-COVID pain exhibited neuropathic features, whereas using the PainDETECT cutoff score of ≥18 points, just 12% of COVID-19 survivors with post-COVID pain had neuropathic features [42]. Still, it should be stressed that neuropathic pain cannot be diagnosed by using self-reported tools only. Instead, per definition, diagnosing or excluding neuropathic pain requires review of the medical record, history taking, and clinical examination (and possibly additional diagnostic examination such as imaging).

Several groups have aimed to identify potential serological findings associated with the presence of neuropathic features in long-COVID patients. Magdy et al. reported higher serum levels of neurofilament light chain in individuals with persistent neuropathic pain symptoms after COVID-19 [43]. On the contrary, no serological biomarker at hospital admission has been associated with development of persistent neuropathic pain after the acute infection [44]. It is hypothesized that long-lasting increased levels of pro-inflammatory biomarkers could facilitate the development of neuropathic pain [43] in agreement with current theories of the SARS-CoV-2 virus [24,25].

It should be noted that none of the abovementioned studies have used objective tests (e.g., electromyography, imaging, or tissue biopsies) for identifying the presence of a neuropathic origin of the pain. A recent case series, including seventeen patients with long COVID, reported that 59% were positive on ≥1 test (e.g., skin biopsy 63%, electrodiagnostic findings 17%, and autonomic function test 50%), confirming neuropathy [45]. Accordingly, the real prevalence of pain of neuropathic origin confirmed with objective measures in individuals with long COVID is still unknown.

### 2.3. Nociplastic Pain

Central sensitization, defined by the IASP as an increased responsiveness of nociceptive neurons in the central nervous system to their normal or subthreshold afferent input [46], is thought to be the main underlying mechanism of nociplastic pain [21]. Other central nervous system-derived symptoms related to neuro-immune alteration, such as fatigue, sleep problems, memory loss, concentration problems, or psychological disturbances, are also typical of nociplastic pain conditions [47] and are often present in individuals with long COVID [4,5,6,7,8,9].

Emerging evidence suggests the presence of central sensitization-associated symptoms in a subgroup of COVID-19 survivors with post-COVID pain. Oguz-Akarsu et al. found that almost 60% of COVID-19 survivors reported multiple pain sites and more than two types of pain symptoms [39]. Ursini et al. observed, through a web-based survey, that 30% of post-COVID pain patients self-reported symptoms compatible with fibromyalgia syndrome [48]. Goudman et al. showed that 70% of subjects with long COVID exhibited sensitization-associated symptoms measured by the Central Sensitization Inventory (CSI) [49,50] (total score ≥ 40/100 points) [51]. Fernández-de-las-Peñas et al. reported a 34% prevalence rate of sensitization-associated symptoms (CSI ≥ 40 points) in another group of patients with post-COVID pain [52]. 

However, current evidence supporting the presence of central sensitization in post-COVID pain is based on self-reported data only. No study has included semi-objective measures of central sensitization, such as quantitative sensory testing or psychophysical testing (e.g., pain thresholds, temporal summation, or conditioned pain modulation testing assessing the functioning of the endogenous pain modulation system). Identification of people with post-COVID pain who exhibit pressure or temperature pain hypersensitivity, impaired temporal summation, or conditioning pain modulation would help to determine the presence of central sensitization in this population. Still, impaired temporal summation or conditioning pain modulation are not specific for patients with nociplastic pain, as they tend to be impaired in those with neuropathic pain too. However, they potentially discriminate between nociceptive and nociplastic pain.

A primary feature of nociplastic pain is the presence of regional or widespread pain symptoms [19]. The presence of regional, including widespread, pain can reach up to 70% of COVID-19 survivors [27,28]. Generalized pain symptoms, combined with central sensitization-associated symptoms (e.g., higher CSI scores) could help identify nociplastic pain in the IASP-established criteria. A recent analysis proposed for phenotyping post-COVID pain supports a model where regional/widespread pain, psychological/emotional disturbance, and other central sensitization-associated symptoms are interconnected, reflecting a nociplastic condition in a subgroup of people with post-COVID pain [53].

## 3. Clinical Criteria/Grading System for Nociplastic Pain in Post-COVID Pain

This section describes the IASP criteria and clinical reasoning process for determining a nociplastic pain phenotype in individuals with post-COVID pain [19] and how to differentiate nociplastic pain from the nociceptive, neuropathic, or mixed phenotypes. Because one patient can fulfill criteria for more than one pain phenotype, it may be most productive to first determine whether nociceptive pain is the predominant pain type. Then, if a nociceptive pain pattern is rejected, additional criteria can be used to differentiate between neuropathic and nociplastic pain.

### 3.1. Step 1—Duration of Pain

An initial requirement for nociplastic pain, according to IASP clinical criteria, is for the patient to report pain symptoms for at least 3 months. It should be noted that the proposed definition for post-COVID-19 syndrome includes the presence of symptoms for at least 2 months post-infection: “Post-COVID-19 condition occurs in people with a history of probable or confirmed SARS-CoV-2 infection, usually 3 months from the onset of COVID-19 with symptoms that last for at least 2 months and cannot be explained by an alternative diagnosis” [2]. In this first step, two demographic features such as age and sex should be considered. For instance, fibromyalgia syndrome, a nociplastic pain condition, has a female predominance. Similarly, female sex has been clearly identified as a risk factor for the development of long COVID symptomatology [54] and also specifically for post-COVID pain [27,28]. The role of age as a risk factor for long COVID is not yet clear [54].

### 3.2. Step 2—Distribution of Pain

To clinically classify nociplastic pain, patients must report a generalized or widespread pain pattern. Secondary to injury, the nociplastic pain extends beyond a specific area of the damaged structure [19]. In contrast, a nociceptive pain pattern is discrete and localized, makes neuroanatomical sense, and can usually be exacerbated with clearly defined pain triggers (specific movements and activities). Accordingly, a careful assessment and interpretation of the patient’s pain distribution is needed. Pain drawings can be used to standardize and optimize the assessment of the individual’s pain distribution in a reliable and valid way [55]. A recent study using pain drawings illustrated that widespread symptoms were present in 20% of COVID-19 survivors exhibiting post-COVID pain [56].

### 3.3. Step 3—Determine Whether Nociceptive Pain Is Present

Another mandatory criterion is that pain cannot be entirely explained by nociceptive mechanisms [19]. Hence, if imaging (such as ultrasonography, X-rays, magnetic resonance imaging (MRI) or computerized tomography (CT) scans) has identified a specific pain generator (e.g., tissue damage) capable of producing nociceptive input that coincides with the patient’s self-reported pain pattern, nociplastic pain can be ruled out as a primary phenotype. If a potential source of nociception is identified that seems likely to be responsible for the post-COVID pain symptoms, the pain should be classified as primarily nociceptive. For instance, as previously stated, COVID-19 survivors can develop localized arthralgias reflecting a potential nociceptive pain mechanism [29,30,31]. It is important to note that identification of a source of nociception in post-COVID pain does not exclude the possibility of concomitant nociplastic or neuropathic pain. This is especially true if pain persists after the source of nociception (e.g., tissue damage) resolves (e.g., tissue damage has healed, but pain remains).

### 3.4. Step 4—Determine Whether Neuropathic Pain Is Present

Similar to nociceptive pain, another mandatory criterion for nociplastic pain states that symptoms cannot entirely be explained by neuropathic pain mechanisms [19]. This includes either diagnosing or refuting neuropathic origin [57] as the dominant post-COVID pain phenotype. According to the IASP definition of neuropathic pain, procedures confirming a lesion or disease of the somatosensory nervous system are mandatory for diagnosis. As previously noted, no study has systematically found evidence of somatosensory nervous system damage in people with post-COVID pain. Development of clinical guidelines for identifying neuropathic pain in this population is clearly needed.

When a neuropathic mechanism is considered to be primarily responsible for post-COVID pain, the pain phenotype should be classified as neuropathic. However, there is a great deal of overlap between neuropathic and nociplastic pain phenotypes, which can make the determination of primary neuropathic pain difficult. Indeed, sustained neuropathic pain can result in increased hyperexcitability of peripheral and central nervous system pain pathways over time [58]. The relationship between neuropathic pain and nervous system sensitization can provide one explanation for the spreading of the pain beyond the innervation territory of the lesioned nervous structure (as with carpal tunnel syndrome) [59], which is consistent with a nociplastic pain phenotype. Thus, it is possible that post-COVID pain patients can exhibit both neuropathic and nociceptive pain patterns (as well as nociplastic pain).

### 3.5. Step 5—Elucidate the Presence of Pain Hypersensitivity

Step 5 involves screening for signs of pain hypersensitivity [19]. This step entails the clinical examination of hyperalgesic (defined as an exaggerated pain response to painful stimuli) and allodynic (defined as pain in response to stimuli that normally do not elicit pain) sensitivity. Nociplastic-related hyperalgesia and allodynia can occur both within the painful region and outside the painful region. Indeed, in patients with nociplastic pain, hyperalgesia and allodynia are often widespread. Clinicians can determine signs of hyperalgesia and/or allodynia with manual palpation or with quantitative sensory testing methods, including pain responses to static and dynamic mechanical or thermal stimuli [60,61]. According to the IASP clinical criteria, if the first five steps are positive for nociplastic pain, a patient can be classified as having “possible nociplastic pain” [19]. If the patient meets criteria in step 6, then pain can be considered “probably nociplastic pain”.

### 3.6. Step 6—Check for History of Pain Hypersensitivity

Step 6 involves examining whether the patient with post-COVID pain reports symptoms of pain hypersensitivity after the infection, which can be assessed by questioning patients about their level of sensitivity to different stimuli. Symptoms of hypersensitivity include pain: when clothing, belts, or jewelry touch or bind one’s skin; a breeze blows on exposed skin; the skin is exposed to water during a bath or shower; a handbag hangs on one’s shoulder; due to pressure on the buttocks while sitting; and during basic activities of daily living.

### 3.7. Step 7—Determine Whether Comorbidities Are Present

The final step involves screening for sensitivity to other stimuli, including sensitivity to sound (phonophobia), light (photophobia), or odors, and the presence of other comorbid symptoms, including poor sleep quality, fatigue, and cognitive problems [19]. As previously documented, all these symptoms are frequently present in individuals with long COVID [4,5,6,7,8,9] and those without post-COVID pain. The CSI can be useful for assessing comorbid symptoms [49,50]. More recently, Tran et al. have developed and published a set of disease-specific PROMs which assess a wide array of post-COVID symptoms that have been reported by long COVID patients [62].

**Figure 1:**
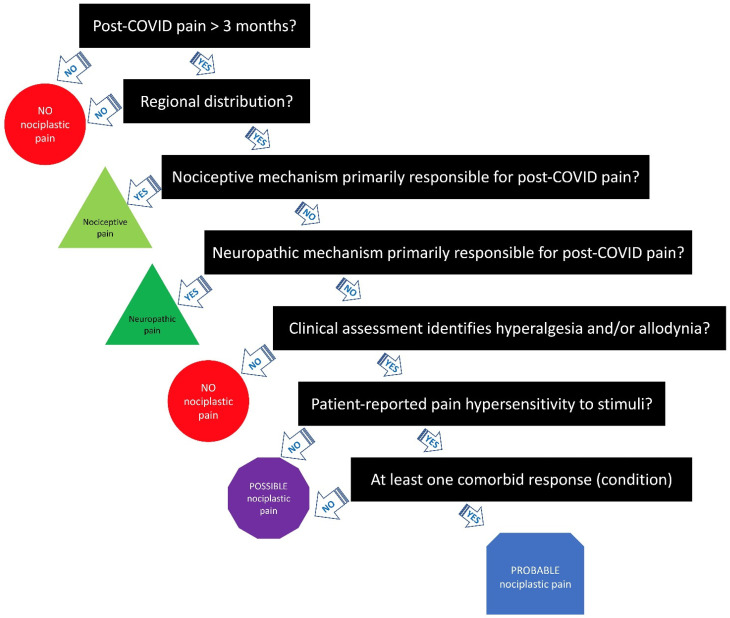
Clinical decision-making tree of the IASP clinical criteria for nociplastic pain applied to post-COVID pain.

If all seven criteria are met, post-COVID pain can be classified as “probable nociplastic pain” [19]. Figure 1 provides a clinical decision-making tree for clinicians who wish to use the IASP clinical criteria for assessing nociplastic pain in people with long COVID. It is important to stress that more research is needed to examine the reliability and validity of the 2021 IASP clinical criteria for nociplastic pain [19] in people with post-COVID pain and other chronic conditions. In fact, it is possible that some patients with post-COVID pain will likely be outside of this phenotype classification, as they will not fall into any of the three categories. In addition, more research is needed to determine the prognostic value and responsiveness of the IASP clinical criteria for nociplastic pain on key treatment outcomes such as pain, function, and health-related quality of life. The treatment responsiveness of the IASP nociplastic criteria [19] in post-COVID pain patients, specifically targeting underlying mechanisms of nociplastic pain such as central sensitization, should be examined in randomized clinical trials. No studies have investigated these proposals.

## 4. Toward Precision Pain Medicine for Post-COVID Pain?

Current knowledge regarding central nervous system sensitization, arguably the main underlying mechanism of nociplastic pain, has resulted in a paradigm shift in the understanding and management of chronic pain conditions [21] and should be directly applied to post-COVID pain. The IASP has provided the first set of clinical criteria, with a grading system linked to nociplastic pain as the third mechanistic pain descriptor (in addition to nociceptive and neuropathic pain) [19]. The application of IASP clinical criteria in long COVID patients will allow clinicians to provide specific treatments according to the pain phenotype. Interestingly, a “musculoskeletal pain cycle” has been recently proposed as a model for guiding therapeutic interventions in chronic musculoskeletal pain conditions [63]. Identification of the predominant pain phenotype in individuals with post-COVID pain will permit the development of a “post-COVID pain” model.

A potential pitfall of this process is that clinicians might neglect the individual variability in one particular pain phenotype. For instance, exercise is a therapeutic strategy recommended for chronic pain and is being proposed as beneficial for individuals with long COVID [64]. In fact, evidence supports that programs combining resistance and aerobic exercises may improve the functional capacity and quality of life (reduce stress or mental disorders) in patients with post-COVID-19 symptoms [65]. However, underlying pain mechanisms must be considered in order to optimize the exercise prescription, especially in people with a nociplastic pain phenotype [66].

This topic is of particular relevance in patients with long COVID, since almost 60% of patients with long COVID report post-exertional malaise (PEM) similar to patients with myalgic encephalomyelitis [67]. In such cases, exercise should be provided with caution, and pacing or other cognitive approaches can be proposed (either in isolation or in combination with exercise therapy). Further, treatment of comorbid symptoms that can perpetuate and interact with pain (e.g., sleep disturbances, fatigue, dyspnea, or autonomic disturbances), especially those with a nociplastic pain phenotype, are essential for optimizing treatment outcomes [68]. In fact, successful outcomes are less likely if treatment is solely focused on improving underlying pain mechanisms (i.e., decreasing central sensitization in the nociplastic post-COVID pain phenotype) without managing associated factors.

It is noted that all known upcoming and current rehabilitation programs for long COVID are focused on aerobic exercise and endurance strategies [69]. Identifying patients with a nociplastic pain phenotype can steer clinicians toward additional treatment approaches, such as pain neuroscience education, cognitive behavioral techniques, or self-regulation/mindfulness strategies, in synergy with exercise. In agreement with this proposal, Bodes-Pardo et al. demonstrated that combining pain neurophysiology education with therapeutic exercise is more effective than application of therapeutic exercise alone in another nociplastic pain condition, chronic lower back pain [70]. Accordingly, multimodal/multifactorial treatment approaches using a biopsychosocial model, which address relevant comorbidities and lifestyle factors for each patient, might amplify the rehabilitation effects for long COVID patients with a nociplastic pain phenotype and produce the most successful treatment outcomes.

## 5. Conclusions

Post-COVID pain remains underestimated and most likely undertreated due to lack of recognition of the phenomenon. Available evidence suggests that nociplastic pain is present in a subgroup of these patients. Applying the global move towards precision medicine to post-COVID pain, and the identification of specific pain phenotypes using the 2021 IASP clinical criteria and grading system [19], could help guide clinical decision making and aid in the most effective treatment planning. The ability of clinicians to phenotype patients with post-COVID pain into nociceptive, neuropathic, nociplastic, or mixed type is important for the following four reasons: (1) proper classification of the pain phenotypes can help clinicians choose proper therapeutic interventions; (2) neuropathic and nociplastic post-COVID pain phenotypes are considered to be more difficult to manage than the nociceptive pain phenotype; (3) to achieve the best treatment outcomes, long COVID patients with nociplastic pain could likely respond best to multimodal treatment approaches to address comorbid symptoms; and (4) the application of mechanism-based treatments may have better clinical outcomes in future clinical trials. Studies examining the clinimetric properties of the 2021 IASP clinical criteria and grading system for nociplastic pain in long COVID patients are needed. Finally, this paper proposes that treatment strategies to be applied to patients with post-COVID pain should be based on pain phenotype and that multimodal approaches should be encouraged. Future trials investigating potential treatment approaches based on the proposed clinical reasoning are now needed.

## Data Availability

Not applicable.
